# Labor market situation after an episode of sickness absence due to malignant neoplasia. Evidence from a Spanish cohort

**DOI:** 10.1186/s12889-019-6792-3

**Published:** 2019-05-03

**Authors:** Ana Lear-Claveras, Monica Ubalde-Lopez, Laura Serra Saurina

**Affiliations:** 1 Center for Research in Occupational Health (CiSAL), Pompeu Fabra University, Barcelona, Spain; 2 IMIM – Parc Salut Mar, Barcelona, Spain; 3 CIBER of Epidemiology and Public Health (CIBERESP), Madrid, Spain

**Keywords:** Disability, Sick leave, Cancer, Labor market, Working life

## Abstract

**Background:**

Due to the progress in screening and cancer treatments, survivor’s prognosis has improved enabling a more likely return to work. However, return to work after a cancer diagnosis may be complex because of an unbalanced health status and work demands relationship that may push them out of the labor market. The aim of this study is to assess the risk of dropping out of the labor market due to unemployment, partial retirement, and permanent disability during the year following an episode due to a malignant neoplasm compared to other non-malignant pathologies.

**Methods:**

Cohort study of 9699 workers affiliated with the Social Security System in Catalonia, who had a sickness absence episode between 2012 and 2013 due to malignant neoplasm, mental, musculoskeletal disorders, cardiovascular diseases and injuries. Competing risk regression models were applied to assess the risk of dropping out of the labor market, by calculating subhazard ratios (SHR) in both sexes. Models were adjusted for age, occupational category, type of contract, economic activity, annual median salary and duration of the SA episode as potential confounders.

**Results:**

Sickness absence due to malignant neoplasia represented 1.7% out of the 9699 episodes included between 2012 and 2013. Although, 80% of individuals continued working in the year following an episode due to malignant neoplasm, women showed a trend towards exiting the labor market because of partial retirement [SHR = 8.4(1.5–45.5)] and permanent disability [SHR = 5.8(1.5–22.9)] compared to non-malignant pathologies. There were no significant differences for unemployment either in women [SHR = 0.4(0.2–0.9)] and in men [SHR = 0.2(0.1–0.6)].

**Conclusions:**

Although return to work is a common pathway among cancer survivors, partial retirement and permanent disability seem to be potential pathways to exit the labor market among women.

## Background

In recent decades the progress of cancer screening and treatment strategies have improved significantly the prognostic of many cancer patients, leading to an increase in the number of survivors and their life expectancy [1, 2]. Workers who have survived over a year after a cancer diagnosis, face problems not only related with clinical aspects of the disease [3], but also with a tough return to work and trouble to continue active in the labor market [4, 5].

According to the European Cancer and Work Network almost half of cancer patients in Europe are diagnosed during working age [1]. Prior research shows that nearly 60% of cancer survivors in working age return to work after treatment [4, 6]. However, as return to work rates depend on cancer location and increases gradually with the time after diagnosis, an effective return to work process is warranted [7]. Overall, 40% of patients had returned to work within the 6 months after diagnosis, this proportion reaches up to 89% at 24 months. Previous evidence shows that the average duration for cancer-related absences varies between 150 and 200 days [4, 8]. Nevertheless, not all cancer survivors are able to continue active in the labor market after the treatment. In some cases, return to work may be complex because of physical, emotional and labor limitations related to the disease, which in turn affect future labor participation over time [6, 9].

Studies focusing on labor trajectories of cancer survivors after dealing with diagnosis and treatment are scarce. Generally, cancer survivors have showed to be 40% more likely to be unemployed in the years following a cancer diagnosis than healthy people [5]. Relevant determinants for future unemployment risk among cancer survivors are location, sex and age, together with unemployment prior the cancer diagnosis, low educational level, manual work, comorbidity or perceived discrimination at the workplace [5, 10, 11]. Moreover, in the years following a cancer diagnosis, survivors are among 40–60% more likely to go on early retirement [12, 13]. Compared to healthy population, cancer survivors have showed to be more likely to have extended or repeated periods of sickness absence (SA) and increased risk for disability pension [14]. Apart from those factors directly related to the disease, old age, low income and low educational level, living alone, having comorbidities and sickness absence benefits in the previous year might also play a role [12–14]. Prior research focused on cancer and labor market participation used dichotomized outcome measures, simplifying the potential consequences of a cancer disease in future working life [11]. In addition, prior evidence is mainly based on cross sectional designs and often using a healthy population as reference group [4, 5]. The aim of the present study is to investigate the risk of an early exit from the labor market due to unemployment, partial retirement, and permanent disability after a sickness absence episode due to a malignant neoplasm compared to other non-malignant diagnoses.

## Methods

### Study design and study population

This is a cohort study based on the linkage of administrative records from two data sources: the Spanish WORKing life Social Security cohort (the WORKss cohort) which provides working life-related data, and the Catalan Institute of Medical Assessment (ICAM) that provides information on the starting/closure date of episodes of sickness absence and medical diagnoses from 2012 to 2014. Briefly, the WORKss cohort is originated from an annual cross-sectional representative sample of a 4% of the Spanish population registered with the Social Security System who had been registered at least one day as workers the year of sampling [15].

The study population (*N* = 9699) consists of salaried workers included in the WORKss cohort between 2012 and 2014, residing in Catalonia and who had the first closed sickness absence episode due to malignant neoplasms and other non-malignant pathologies (i.e. mental, musculoskeletal disorders, cardiovascular diseases and injuries) between 2012 and 2013. Individuals were followed from the SA episode closure date until the exit of the labor market due to unemployment, partial retirement, permanent disability, death or end of the follow up.

### Variables

Unemployment, partial retirement and permanent disability status were identified as a way to drop out the labor market in the year following a sickness absence episode between 2012 and 2014. Diagnosis for the first sickness absence episode, coded with the International Classification of Diseases 10th version (ICD-10), was the main independent variable. Diagnoses were classified into two groups: 1) malignant neoplasms [ICD-10: C00-C97] (*n* = 167), and 2) other non-malignant pathologies [mental disorders (ICD-10: F00-F99), musculoskeletal disorders (ICD-10: M00-M99), cardiovascular diseases (ICD-10: I00-I99) and injuries (ICD-10: S00-T88)] (*n* = 9532).

Age (classified into four groups: ≤30, 31–45, 46–55 and > 55 years), occupational category (skilled non-manual, skilled manual, unskilled non-manual and unskilled manual), type of contract (permanent, temporary), economic activity (agriculture, industry, construction, services and extraterritorial organisms- i.e., international organizations, excluding consular offices located in the foreigner), annual median salary (below or above the median annual salary) and the SA episode duration, were considered as potential confounders for men and women separately.

### Statistical analysis

A descriptive analysis summarizing categorical variables as frequencies and continuous variables by means of central tendency measures, was performed to estimate the distribution of SA episodes. SA duration was expressed as the median duration (MD) in days, 25th (P25) and 75th (P75) percentiles.

Competing risk regression models were applied to assess the risk of dropping out of the labor market, for individuals who had a SA episode due to malignant neoplasms compared to those who had episodes because other non-malignant pathologies. Subhazard ratios (SHR) with 95% confidence intervals (95%CI) were calculated in both sexes. [16]. The competing risk regression models were adjusted for age, occupational category, type of contract, economic activity, annual median salary and duration of the SA episode. For partial retirement only individuals older than 55 were included. Deaths occurred before the events of interest (i.e., unemployment, partial retirement and permanent disability) were censored. Statistical analysis was carried out using StataCorp. 2011. Stata: Release 12. Statistical Software. College Station, TX: StataCorp LP.

## Results

A total of 9699 first closed SA episodes due to target diagnoses were registered between 2012 and 2013, 54% in women. Malignant neoplasms represented around 1.7 and 1.8% in women and men respectively.

Episodes due to malignant neoplasms showed the longer durations in both sexes (MD: 29 days in women and 24 days in men); followed by mental disorders (MD: 18 days in women and 19 days in men) and cardiovascular diseases (MD: 18 days in women and 17 days in men). The most common malignant neoplasms diagnoses were genitourinary cancer among men, and breast cancer for women [Table 1].

**Table 1:** Distribution of sickness absence episodes due to target diagnosis groups among Spanish salaried workers, 2012–2013

	Women		Men	
Diagnostic groups (ICD-10)	1^rst^ closed episodes	SA days p50 (p25–p75)	1^rst^ closed episodes	SA days p50 (p25–p75)
Malignant neoplasms	88 (1.7)	29 (12–95)	79 (1.8)	24 (12–67)
Genitourinary organs (C51.0-C68.9)	15 (17)	27 (9–116)	37 (46.8)	25 (19–60)
Breast (C50-C50.9)	40 (45.4)	44 (12–110)	–	–
Skin (C43-C44.9)	14 (16)	16 (11–24)	10 (12.6)	10 (5–26)
Respiratory system (C30-C39.9)	–	–	9 (11.4)	32 (9–106)
Other malignant neoplasms	19 (21.6)	37 (13–189)	23 (29.2)	15 (8–135)
Mental disorders	981 (18.6)	18 (8–48)	553 (12.4)	19 (8–48)
Cardiovascular disease	273 (5.2)	18 (9–37)	336 (7.6)	17 (7–36)
Musculoskeletal system	2927 (55.6)	14 (5–37)	2116 (47.7)	9 (5–23)
Injuries	992 (18.9)	16 (7–42)	1354 (30.5)	15 (7–38)
Total	5261 (100)	15 (6–40)	4438 (100)	12 (5–33)

Return to work is the most common pathway after a SA episode due to malignant neoplasm (78% women, 85% men). Overall, there were 2053 episodes of unemployment (54% among women), 31 individuals who took partial retirement, 64 entered on permanent disability, and 18 died during the follow up. In both sexes, unemployment episodes occurred mainly among young workers (more than 70% in workers under 45 years), with permanent contracts (more than 50% in both sexes), employed in the service sector (about 89 and 64% in women and men respectively) and with low salaries (81% women, 62% men). The higher proportion of partial retirement was observed in temporary workers (more than 75% in both sexes), employed in the service sector (79% women, 47% men), with high salaries (86% women, 76% men), in unskilled non-manual (43% women) and skilled manual (53% men). Permanent disability was mainly common among workers over 45 years (more than 80% in women, 70% in men), with permanent contracts (67% women, 71% men), employed in the service sector (90% women, 59% men) and in unskilled non-manual (37% women) and skilled manual occupations (59% men) [Table 2].

**Table 2:** Episodes of unemployment, partial retirement and permanent disability in the year following a sickness absence episode^a^

		Women (*n* = 5261)			Men (*n* = 4438)		
		1^rst^ Unemployment	Partial retirement	Permanent disability	1^rst^ Unemployment	Partial retirement	Permanent disability
		n (%)	n (%)	n (%)	n (%)	n (%)	n (%)
Age							
< 30		284 (25.5)	–	–	232 (24.7)	–	1 (2.9)
31–45		536 (48.1)	–	5 (16.7)	436 (46.4)	–	9 (26.5)
46–55		195 (17.5)	–	10 (33.3)	171 (18.2)	–	9 (26.5)
> 55		99 (8.9)	14 (100)	15 (50.0)	100 (10.7)	17 (100)	15 (44.1)
Type of contract							
Permanent		651 (58.5)	3 (21.4)	20 (66.7)	505 (53.8)	3 (17.6)	24 (70.6)
Temporary		455 (40.8)	11 (78.6)	6 (20.0)	421 (44.8)	13 (76.5)	9 (26.5)
Not include		8 (0.7)	–	4 (13.3)	13 (1.4)	1 (5.9)	1 (2.9)
Occupational category							
Skilled non-manual		124 (11.1)	5 (35.7)	5 (16.7)	85 (9.0)	3 (17.7)	2 (5.9)
Unskilled non-manual		509 (45.7)	6 (42.9)	11 (36.7)	202 (21.5)	4 (23.5)	8 (23.5)
Skilled manual		255 (22.9)	3 (21.4)	7 (23.3)	471 (50.2)	9 (52.9)	20 (58.8)
Unskilled manual		218 (19.6)	–	6 (20.0)	181 (19.3)	1 (5.9)	4 (11.8)
Not include		8 (0.7)	–	1 (3.3)	–	–	–
Economic activity							
Agriculture		3 (0.3)	–	1 (3.3)	10 (1.0)	–	1 (2.9)
Industry		94 (8.4)	3 (21.4)	2 (6.7)	152 (16.2)	7 (41.3)	10 (29.4)
Construction		13 (1.2)	–	–	163 (17.4)	1 (5.8)	3 (8.8)
Services		996 (89.4)	11 (78.6)	27 (90.0)	602 (64.1)	8 (47.1)	20 (58.8)
Extraterritorial organisms		–	–	–	–	–	–
Missing		8 (0.7)	–	–	12 (1.3)	1 (5.8)	–
Annual median salary							
< median		907 (81.4)	2 (14.3)	22 (73.3)	581 (61.9)	4 (23.5)	20 (58.8)
> median		205 (18.4)	12 (85.7)	7 (23.3)	358 (38.1)	13 (76.5)	14 (41.2)
Missing		2 (0.2)	–	1 (3.3)	–	–	–
Total		1114 (100)	14 (100)	30 (100)	939 (100)	17 (100)	34 (100)

^a^Episodes due to malignant neoplasms and other non-malignant pathologies (i.e., cardiovascular diseases, musculoskeletal and mental disorders, and injuries

**Fig. 1:**
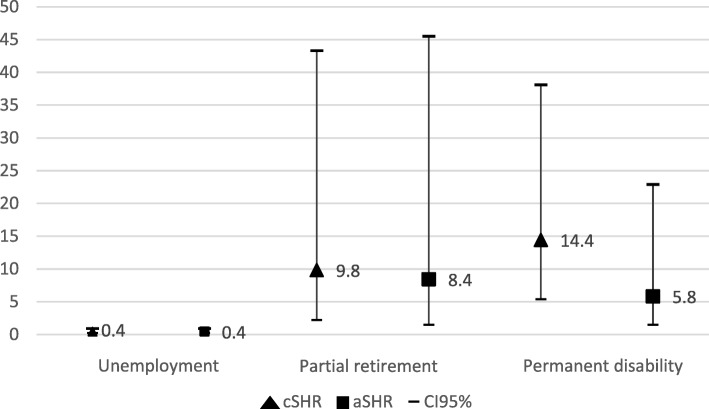
Risk to exit the labor market in the year following a sickness absence episode due to malignant neoplasm among women. Reference category: other non-malignant pathologies (i.e.; cardiovascular diseases, musculoskeletal and mental disorders, and injuries); cSHR: crude Sub-Hazard Ratio; aSHR: adjusted Sub-Hazard Ratio for age (unemployment and permanent disability), type of contract, occupation category, economic activity, annual median salary and duration of the sickness absence episode

**Fig. 2:**
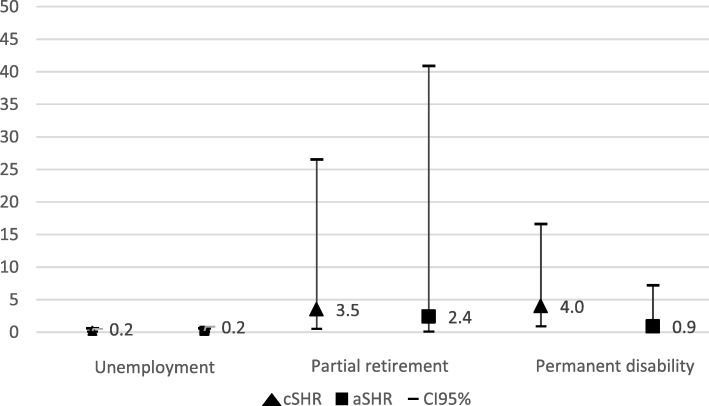
Risk to exit the labor market in the year following a sickness absence episode due to malignant neoplasm among men. Reference category: other non-malignant pathologies (i.e.; cardiovascular diseases, musculoskeletal and mental disorders, and injuries); cSHR: crude Sub-Hazard Ratio; aSHR: adjusted Sub-Hazard Ratio for age (unemployment and permanent disability), type of contract, occupation category, economic activity, annual median salary and duration of the sickness absence episode

The competing risk regression models showed a lower risk of dropping out the labor market because of unemployment for those who had episodes due to malignant neoplasms (aSHR = 0.4; CI95% 0.2–0.9 for women; aSHR = 0.2; CI95% 0.1–0.6 for men). Women showed a trend towards exiting the labor market because of partial retirement (aSHR = 8.4; CI95% 1.5–45.4) and permanent disability (aSHR = 5.8; CI95% 1.5–22.9) compared to non-malignant pathologies [Fig. 1]. We did not observe significant results for men [Fig. 2].

## Discussion

To our knowledge, this is the first study that investigates jointly the risk of early dropping out of the labor market due to unemployment, partial retirement and permanent disability after a sickness absence episode due to a malignant neoplasm compared to other non-malignant diagnoses.

In our study the most women and men who had a SA due to malignant neoplasm continue working within the year after the episode. Our results are consistent with previous studies showing return to work rates between 25% and over 90% [4, 6, 7], according to cancer site, stage or type of treatment, the time after the end of treatment or symptoms related to the disease [17, 18].

Compared to other non-malignant pathologies, we did not find unemployment as a pathway to drop out the labor market after a malignant neoplasm. This result is inconsistent with prior findings showing cancer survivors to have 40% more risk to be unemployed. A prior study on breast cancer survivors, found duration of prior unemployment as the main determinant for future unemployment [11]. Nevertheless, most prior studies have used a healthy population as a reference group [4–6, 17] instead of a working population that were on sickness absence. On the other hand, our results suggest that women who had a malignant neoplasm are more likely to exit the labor market because of partial retirement and permanent disability that those with other pathologies. These results are in line with prior studies that have assessed the risk of dropping out comparing with healthy working populations [12–14, 19, 20]. Compared to other disabling diagnoses (e.g. musculoskeletal disorders), malignant neoplasms may result in health-related problems and functional (physical and mental) limitations, due to its severity and treatment, that might reduce considerably working ability making unfeasible to continue active in labor market.

Partial retirement could be considered as a workplace adjustment by reducing working hours. Previous studies showed that one in four of cancer survivors who continue in the labor market experience a deterioration of their physical and mental capacity to work and need to make work adjustments [21]. Reducing or adapting the work schedule are among the most common worksite adjustment strategies [21–25]. Reducing working hours and tailoring work tasks, their functional capacity and health status, could improve their own perception and expectations about their ability to perform at work, facilitating their continuity in the labor market [26].

One of the strongest predictors to return to work is workers’ own perception and expectations once back to work [4, 21, 27]. Factors related to work and employment conditions, work environment and social support from colleagues and supervisors also determine the return to work as it could modify the perception of their capacity to perform at work [4, 17, 21, 28], hampering the return to work process, or reducing their expectations to continue engaged in the labor market after a malignant neoplasm [26, 29]. Previous studies suggest that keeping contact with coworkers during treatment and maintaining a positive relationship with them could facilitate the continuity in the labor market [4, 26].

Our results must be interpreted with caution considering some limitations. It is possible that a year of follow-up after the first SA episode is insufficient to measure the long-term effect of the disease on continuing active into the labor market. In the case of unemployment, it is feasible to be a later option for those who returned to work after a SA episode, therefore we could be underestimating the risk. Previous studies show that of the total of cancer survivors who returned to work during the first year, 11% stop working for reasons related to the disease in the next 3 years [29]. This might be also the case for partial retirement and permanent disability. Eligibility for a benefit from a disability pension comprises a complex and long-lasting process, and requires a minimum time having paid into the social security system. Therefore, we might consider that workers with temporary contracts may not have accumulated enough time to qualify for those benefits and, in turn, they had no access to permanent disability pension. In addition, it is possible there were cases already enrolled into the permanent disability recognition process during the year of follow-up. In Spain, permanent disability pension allows to return to work, to a different occupation of the former one at the time of diagnosis, depending on the degree of disability. Therefore, it is possible that some workers had returned to work after the follow up period. Likewise, we had no available information about interventions potentially carried out in the workplace aimed to facilitate the return to work.

Finally, we had no information related to health status or aspects related to the disease, such as the stage, type of treatment, side effects or the most frequent complications that could reduce the work functioning or to fully incapacitate. However, we have included the duration of the SA episode as measure of prior health status as a proxy for the severity of the pathology. Nevertheless, our study population belongs to a large representative sample of the Spanish working population affiliated to the Social Security System, and covers a wide range of economic activities and occupations, which strengthen the external validity of the results. Moreover, data comes from reliable administrative records, which allows to avoid memory biases and reduces associated costs.

## Conclusions

Although the most workers who had a SA due to malignant neoplasm continue working within the year after the episode, health problems and functional limitations related to the disease and their treatment have an impact in the future labor participation. Among women who survived a cancer, partial retirement and permanent disability might be potential pathways to exit the labor market, probably due to an unbalanced functional limitation and job demands relationship.

Our study contributes to expand the knowledge around the labor market participation of cancer survivors after a SA episode and potential pathways to exit the labor market. Given the increase in life expectancy of cancer survivors in working ages, it would be advisable to promote actions that adapt and their work ability. Further investigation on determinants of labor market participation is needed in order to promote tailored workplace interventions looking at adaptations that promote a healthy and safe working life and retain cancer survivors active into the labor market.

## Abbreviations

95%CI: Confidence interval
aSHR: Adjusted subhazard ratio
cSHR: Crude subhazard ratio
ICD-10: International Classification of Diseases 10th version
MD: Median duration
P25: 25th percentile
P75: 75th percentile
SA: Sickness absence
WORKss cohort: WORKing life Social Security cohort

## References

[1] de Boer AG (2014). The European Cancer and work network: CANWON. J Occup Rehabil.

[2] Ferlay J, Autier P, Boniol M, Heanue M, Colombet M, Boyle P (2007). Estimates of the cancer incidence and mortality in Europe in 2006. Ann Oncol.

[3] Harrington CB, Hansen JA, Moskowitz M, Todd BL, Feuerstein M (2010). It's not over when it's over: long-term symptoms in cancer survivors--a systematic review. Int J Psychiatry Med.

[4] Mehnert A (2011). Employment and work-related issues in cancer survivors. Crit Rev Oncol Hematol.

[5] de Boer AG, Taskila T, Ojajärvi A, van Dijk FJ, Verbeek JH (2009). Cancer survivors and unemployment: a meta – analysis and meta – regression. JAMA.

[6] Taskila T, Lindbohm ML (2007). Factors affecting cancer survivors’ employment and work ability. Acta Oncol.

[7] Roelen CA, Koopmans PC, Groothoff JW, van der Klink JJ, Bültmann U (2011). Sickness absence and full return to work after cancer: 2-year follow-up of register data for different cancer sites. Psychooncology..

[8] Delclós J, García S, López JC, Sampere M, Serra C, Plana M (2010). Duration of non-work related sickness absence by clinical diagnosis. Arch Prev Riesgos Labor.

[9] Roelen CA, Koopmans PC, Groothoff JW, van der Klink JJ, Bültmann U (2011). Return to work after cancer diagnosed in 2002, 2005 and 2008. J Occup Rehabil.

[10] Carlsen K, Dalton SO, Diderichsen F, Johansen C (2008). Risk for unemployment of cancer survivors: a Danish cohort study. Eur J Cancer.

[11] Carlsen K, Ewertz M, Dalton SO, Badsberg JH, Osler M (2014). Unemployment among breast cancer survivors. Scand J Public Health..

[12] Sesto ME, Faatin M, Wang S, Tevaarwerk AJ, Wiegmann DA (2013). Employment and retirement status of older cancer survivors compared to non-cancer sibling. WORK.

[13] Carlsen K, Dalton SO, Frederiksen K, Diderechsen F, Johansen C (2008). Cancer and the risk for taking early retirement pension: a Danish cohort study. Scand J Public Health.

[14] Nord C, Olofsson S, Glimelius I, Cedermark GC, Ekberg S, Cavallin-Ståhl E (2015). Sick leave and disability pension among Swedish testicular cancer survivors according to clinical stage and treatment. Acta Oncol.

[15] López Gómez MA, Durán X, Zaballa E, Sanchez-Niubo A, Delclós J, Benavides FG (2016). Cohort profile: the Spanish WORKing life social security (WORKss) cohort study. BMJ Open.

[16] Gutierrez RG (2010). Competing-risks regression. Stata corporation LP, Boston conference.

[17] Spelten ER, Sprangers MA, Verbeek JH (2002). Factors reported to influence the return to work of cancer survivors: a literature review. Psychooncology.

[18] Spelten ER, Verbeek JH, Uitterhoeve AL, Ansink AC, van der Lelie J, de Reijke TM (2003). Cancer, fatigue and the return of patients to work - a prospective cohort study. Eur J Cancer.

[19] Hauglann BK, Saltyte Benth J, Fossa SD, Tveit KM, Dahl AA (2014). A controlled cohort study of sickness absence and disability pension in colorectal cancer survivors. Acta Oncol.

[20] Lindbohm ML, Kuosma E, Taskila T, Hietanen P, Carlsen K, Gudbergsson S (2014). Early retirement and non-employment after breast cancer. Psychooncology..

[21] Torp S, Nielsen RA, Gudbergsson SB, Dahl AA (2012). Worksite adjustments and work ability among employed cancer survivors. Support Car Cancer.

[22] Ell K, Xie B, Wells A, Nedjat-Haiem F, Lee PJ, Vourlekis B (2008). Economic stress among low-income women with cancer: effects on quality of life. Cancer..

[23] Moran JR, Short PF, Hollenbeak CS (2011). Long-term employment effects of surviving cancer. J Health Econ.

[24] Steiner JF, Cavender TA, Nowels CT, Beaty BL, Bradley CJ, Fairclough DL (2008). The impact of physical and psychosocial factors on work characteristics after cancer. Psychooncology..

[25] Syse A, Tretli S, Kravdal O (2008). Cancer's impact on employment and earnings--a population-based study from Norway. J Cancer Surviv.

[26] Wells M, Williams B, Firnigl D, Lang H, Coyle J, Kroll T (2013). Supporting “work-related goals” rather than “return to work” after cancer? A systematic review and meta-synthesis of 25 qualitative studies. Psychooncology..

[27] de Boer AG, Verbeek JH, Spelten ER, Uitterhoeve AL, Ansink AC, de Reijke TM (2008). Work ability and return-to-work in cancer patients. Br J Cancer.

[28] Sampere M, Gimeno D, Serra C, Plana M, Lopez JC, Martínez JM (2012). Return to work expectations of workers on long-term non-work-related sick leave. J Occup Rehabilb.

[29] Short PF, Vasey JJ, Tunceli K (2005). Employment pathways in a large cohort of adult cancer survivors. Cancer..

